# Neutrophil extracellular traps (NETs)-mediated killing of carbapenem-resistant hypervirulent *Klebsiella pneumoniae* (CR-hvKP) are impaired in patients with diabetes mellitus

**DOI:** 10.1080/21505594.2020.1809325

**Published:** 2020-08-29

**Authors:** Longyang Jin, Yudong Liu, Chendi Jing, Ruobing Wang, Qi Wang, Hui Wang

**Affiliations:** Department of Clinical Laboratory, Peking University People’s Hospital, Beijing, China

**Keywords:** Neutrophil extracellular traps (NETs), carbapenem-resistant hypervirulent *Klebsiella pneumoniae* (CR-hvKP), type 2 diabetes mellitus (T2D), antimicrobial activity, innate immunity

## Abstract

Carbapenem-resistant hypervirulent *Klebsiella pneumoniae* (CR-hvKP) have been reported in recent years across Asian countries and pose a serious threat to public health. Neutrophils represent the first line of defense against numerous infectious pathogens, such as CR-hvKP. Neutrophil extracellular traps (NETs) constitute one of the major antimicrobial defense mechanisms in neutrophils against invading pathogens, especially against hvKP. Interestingly, previous studies have demonstrated that patients with type 2 diabetes mellitus (T2D) display elevated levels of NETosis but are vulnerable to infections caused by hvKP. The discrepancy propels us to investigate the role of NETs in hvKP infections in the context of T2D. By utilizing a clinical-derived CR-hvKP strain and a combination of NETs complex detection, phagocytosis testing, NETs killing assay and immunofluorescence, and scanning electron microscope assays, we identified defective NETs-mediated killing of CR-hvKP strain in patients with T2D. Specifically, we show that the impaired NETs-mediated killing in T2D is not due to the decreased NETs formation, as the neutrophils isolated from T2D patients exhibited enhanced NETs formation compared to healthy controls. Further, we demonstrate that the reduced NETs activity does not result from the trapping failure of CR-hvKP, but likely associated with the deficient surface damage conferred by the NETs of T2D patients. Our data provide a novel insight into the defective innate immune response against CR-hvKP in T2D.

## Introduction

*Klebsiella pneumoniae* has become notorious worldwide and represents major cause of hospital-associated infections, including pneumonia, intra-abdominal infection, bloodstream infection, and urinary tract infection. Carbapenem-resistant *K. pneumoniae* (CRKP), which exhibit resistance to almost all available antimicrobial agents, have rapidly increased worldwide, which are largely due to successful spread and transmission of CRKP prevalent clones. Sequence type 11 (ST11) CRKPs have been shown as the most dominant clone in China, accounting for up to 75% of CRKPs [1,2]. ST11 CRKP strains are associated with a substantial increase in morbidity and mortality, particularly in the immunosuppressive patients [1]. Even worse, ST11 CRKPs are able to obtain a pLVPK-like virulence plasmid from hypervirulent *K. pneumoniae* (hvKP) and evolve into CR-hvKP [3]. In fact, ST11 CR-hvKPs are associated with a fatal ventilator-associated pneumonia outbreak in an intensive care unit [3] and have been shown widely disseminated across the China [4]. The real “superbug” with simultaneous high pathogenicity and multidrug-resistance pose an urgent threat to public health, especially for people with underlying diseases.

Type 2 diabetes mellitus (T2D) is an important metabolic disease characterized by dysregulated immune responses and insulin resistance. Over the past decades, the prevalence of T2D steadily rises in both developing and developed countries [5]. Clinical observations suggest a significant link between diabetes mellitus and an increased susceptibility to infections, especially to *K. pneumoniae*. In addition, increasing evidence has demonstrated that patients with T2D have higher risk for hvKP infection [6–8]. Remarkably, 75% patients with liver abscess due to hvKP had underlying diabetes or glucose intolerance [9]. Poor glycemic control also has been associated with significantly more metastatic spread, which is one of the typical syndromes of hvKP infection [6]. Hypothesized mechanisms include increased serum glucose levels, loss of vascular patency or integrity, and the persistent inflammatory state in this patient group [6]. However, the molecular basis of the propensity of hvKP infection to diabetic patients has not been well understood and required further investigation.

The recent emergence of CR-hvKP, which are CRKPs equipped with hypervirulence, further complicated the clinical treatment. Innate immune arm, particularly neutrophils, represents the first line of host defense against CR-hvKP. Neutrophil extracellular traps (NET) is an extracellular web-like decondensed chromatin network decorated by neutrophil-derived proteins, including enzymes, antimicrobial peptides, calgranulin, and histones [10]. Many factors, such as phorbol 12-myristate 13-acetate (PMA), lipopolysaccharides (LPS), and calcium ionophore, can induce NETs formation [11]. In addition, numerous microbes, including hvKP, are also able to induce NETosis [12,13]. During hvKP infection, NETs play a protective role through their capacity to trap, inhibit, and kill the invading bacteria [12,13].

However, the mechanism through which NETs affect antimicrobial defense in the context of hvKPs in T2D remains largely unknown. Previous study indicated that T2D impairs NET formation, thereby leading to delayed release of short-lived and unstable NETs [14], which may explain why patients with T2D are more vulnerable to hvKPs infections. Interestingly, recent evidence suggested that NETs are overproduced in T2D, as shown by enhanced circulating levels of NET-associated proteins, including citrullinated histone H3 (Cit-H3) and DNA-MPO complex, in patients with diabetes [15,16]. In addition, neutrophils from patients with T2D as well as murine model of diabetes mellitus are primed to produce NETs [15]. In this case, a particularly intriguing question is raised why patients with T2D produce excess NETs but are more susceptible to hvKPs infection. In the present study on a Chinese T2D cohort, we confirmed that patients with T2D are more readily to undergo NETosis upon stimulation. However, using a clinical-derived hvKP strain that belongs to the prevalent ST11 type of CRKP in China, we proposed that the decreased surface damage conferred by NETs of T2D patients was a possible reason for the impaired NETs killing activity. Our findings revealed that the impaired NETs-mediated bactericidal capacity may contribute to the increased susceptibility of patients with T2D to hvKPs infections. Our data have important clinical implications regarding NETs-based therapies against CR-hvKP infections in T2D.

## Methods

### Bacterial strain

Clinical *K. pneumoniae* isolate C2166 was collected from a blood sample of an inpatient, who died of sepsis and antibiotic treatment failure in Peking University People’s Hospital in 2017. Antimicrobial susceptibility tests were performed using the broth microdilution method by following the Clinical and Laboratory Standards Institute guidelines [17]. Hypervirulence traits were determined by clinical outcome, *in vitro* serum killing, and *Galleria mellonella* lethality assays, as described previously [4]. Multilocus sequence and capsule typing, antimicrobial resistance genes and virulence genes were identified by whole genome sequencing using PacBio Sequel system. The draft genome of C2166 was assembled using Canu 2.0 and comprised 7 contigs in total and 5 contigs containing plasmid replicons (IncFIB, IncFII/IncR, IncHI1B, and two ColRNAI). The sequence data have been deposited in GenBank (accession number JABXOA000000000).

### Subjects

A total of 20 patients with T2D were included in the study. The diagnosis of T2D was in accordance with the World Health Organization criteria [18]. Only those with HbA1c (glycated hemoglobin/hemoglobin A1c) greater than 8.5% were included in this study [19]. A total of 20 age- and gender-matched healthy individuals were enrolled as healthy controls (HCs). HCs were defined as no signs of infection or inflammation or other significant illnesses.

### NETs complex detection

NETs were quantified by an ELISA detecting MPO-DNA complexes, as previously described [20,21]. Briefly, polyclonal rabbit anti-human MPO antibody (Cat # A039829-2, DAKO) was coated in 96-well plate overnight at 4°C. After three washes with washing buffer (PBS with 0.05% Tween 20), 200 μl blocking buffer were added and incubated for 2 h at room temperature. After washing procedure, 100 μl of diluted serum samples were added to the wells. The plate was incubated for 2 h at room temperature. After three washes, anti-dsDNA mouse monoclonal antibody (Cat # MAB030, Sigma-Aldrich) in 100 μl blocking buffer were added and incubated for 2 h at room temperature. Peroxidase-labeled goat anti-mouse IgG secondary antibody (Cat # 1706516, Bio-Rad), peroxidase substrate TMB (Cat # C520026, Sangon Biotech) and 1 M H_2_SO_4_ were applied to detect absorbance at 450 nm. Specific OD was obtained by subtracting total OD from background OD generated without the addition of peroxidase-labeled secondary antibody.

### In vitro NETs induction

Neutrophils were prepared as described previously [12]. Briefly, blood samples were collected from HCs and patients with T2D and used immediately. Neutrophils were isolated from peripheral blood by density gradient centrifugation on Ficoll Paque (Cat # 17144002, GE Healthcare) according to the manufacturer’s instructions. Erythrocytes were sedimented using dextran and further lysed by ACK lysing buffer (Cat # A1049201, Thermo Fisher). The purity of the neutrophils was above 95%. Subsequently, neutrophils were washed with PBS and suspended in phenol red-free RPMI 1640 at a density of 5 × 10^6^ cells/mL. Isolated neutrophils were seeded on poly-L-lysine-coated glass coverslips (Cat # 1254580, Fisher Scientific) in 24-well plates and allowed to settle onto the coverslip for 0.5 h. NET inducers, PMA (Cat # ab120297, Abcam), and calcium ionophore A23187 (Cat # C7522, Sigma-Aldrich), were added to stimulate neutrophils to release NETs. After 3 h, cells were fixed with paraformaldehyde overnight at 4°C.

### NETs staining and visualization

NETs staining was performed as previously described [20,21]. Briefly, fixed neutrophils were blocked with blocking buffer (0.2% gelatin in PBS, Cat # G1890, Sigma-Aldrich) at 37°C for 1 h. Then, cells were stained using anti-human MPO primary antibody (Cat # A039829-2, DAKO) at 37°C for 2 h in a humid chamber. After three washes with PBS, the cells were incubated for another 1 h with donkey anti-rabbit IgG secondary antibody conjugated with Alexa Fluor 555 (Cat # A31572, Thermo Fisher). The DNA was stained with Hoechst 33342 (Cat # H3570, Thermo Fisher). NETs were visualized on a Leica TCS SP8 confocal microscope and low magnification images (40×) were acquired with software on non-overlapping random images (6 separate fields/coverslips). NETs were manually quantitated on acquired images as Hoechst-positive fibrils released by single neutrophil with an overall length at least twice as long as the cell diameter and expressed as percentage of neutrophils with released DNA. Each experiment was conducted in triplicate.

### Neutrophil killing assays

Isolated neutrophil (1 × 10^6^ cells) were combined with serum-opsonized bacteria (1 × 10^6^ CFUs) in 2-mL Eppendorf tubes with a final volume of 200 μL and were incubated at 37°C for 60 min with gentle rotation. NETs formation of neutrophils were inhibited by pre-incubating for 1 h in the presence of DNase I. Cells were subsequently lysed with 0.1% Triton X-100 on ice for 15 min and viable bacteria were quantified by plating serial dilutions on LB agar. As controls, bacterial growth in RPMI 1640 media without neutrophils at the 60-min time point was also quantified. Survival was expressed as the percentage of CFUs recorded after neutrophil treatment compared with the control. All experiments were done in triplicate.

### Neutrophil phagocytosis assay

Fluorescein isothiocyanate (FITC) labeled bacteria were prepared as described previously [22]. Briefly, the bacteria in the exponential phase firstly were heat killed for 60 min in a 70°C water bath. After washed with PBS, the bacteria were labeled with FITC (Cat # F4274, Sigma-Aldrich) by incubation with 0.1 mg/mL FITC in 0.1 M NaHCO_3_, pH 9.0, for 60 min at 25°C. Bacteria were then washed of unbound fluorochrome with PBS by three cycles of centrifugation. FITC-labeled *E. coli* ATCC 25922 and *K. pneumoniae* strain C2166 were analyzed by flow cytometry (FACSCanto, BD) to ensure the percentage of FITC-positive bacteria was greater than 95%. Isolated neutrophil (1 × 10^6^ cells), FITC labeled bacteria (4 × 10^7^ CFUs), 10% normal human serum were mixed and suspended in PBS to final volume of 1.0 mL. Each sample was incubated at 37°C with continuous agitation. After incubation of 0, 20, and 40 min, the suspension was transferred to an ice bath immediately. The superficial fluorescent was quenched by adding 100 μl Trypan Blue (0.04%) to determine the percentage of neutrophils with ingested bacteria. The percentage of neutrophils ingested bacteria was assessed by flow cytometer (FACSCanto, BD). A total of 50,000 neutrophils were processed for each sample. Phagocytosis rate was determined by the percentage of FITC positive neutrophils.

### NETs-mediated killing

Isolated neutrophils (1 × 10^6^ cells) were suspended in 500-μL RPMI 1640 and seeded in 24-well plates to generate NETs induced by 100 nM PMA. After 3 h incubation at 37°C, C2166 (1 × 10^6^ CFUs) were added to appropriate wells and incubated for another hour. Cells were subsequently lysed with 0.1% Triton X-100 on ice for 15 min and C2166 were plated on LB agar. Colonies were enumerated the following day and percentage survival was calculated with the control.

### Scanning electron microscope observations

As described above, C2166 were co-cultured for 1 h with PMA induced neutrophils isolated from healthy individuals and diabetes subjects. Subsequently, NETs and bacteria were fixed for 24 h at 4°C with 2.5% glutaraldehyde. After standard dehydration and sputter coating process, NETs and bacteria surface were visualized under a scanning electron microscope (JSM-7900F, JEOL) at an acceleration voltage of 2.0 kV. Ten to 15 NETs-bacteria complex fields were selected randomly and over 150 bacteria were counted and analyzed. Clear alterations of the cell surface structure, such as change from smooth to rough, decreased surface stiffness, formation of micelles and blisters, indicated a bacterial surface damage during the interaction with NETs complex. This experiment was performed in triplicate.

### Statistical analysis

All experiments were performed in triplicate, and statistical analyses were performed with SPSS 17.0 software (SPSS Inc., Chicago, IL, USA). Statistical significance was determined using Student’s t-test and defined as *p* < 0.05.

### Study approvals

Human blood samples collection was approved by the Ethics Committee of Peking University People’s Hospital and performed in accordance with the ethical standards of Declaration of Helsinki of 1975. Informed consent was waived as all data were anonymized.

## Results

### NETosis is enhanced in T2D

**Figure 1.**
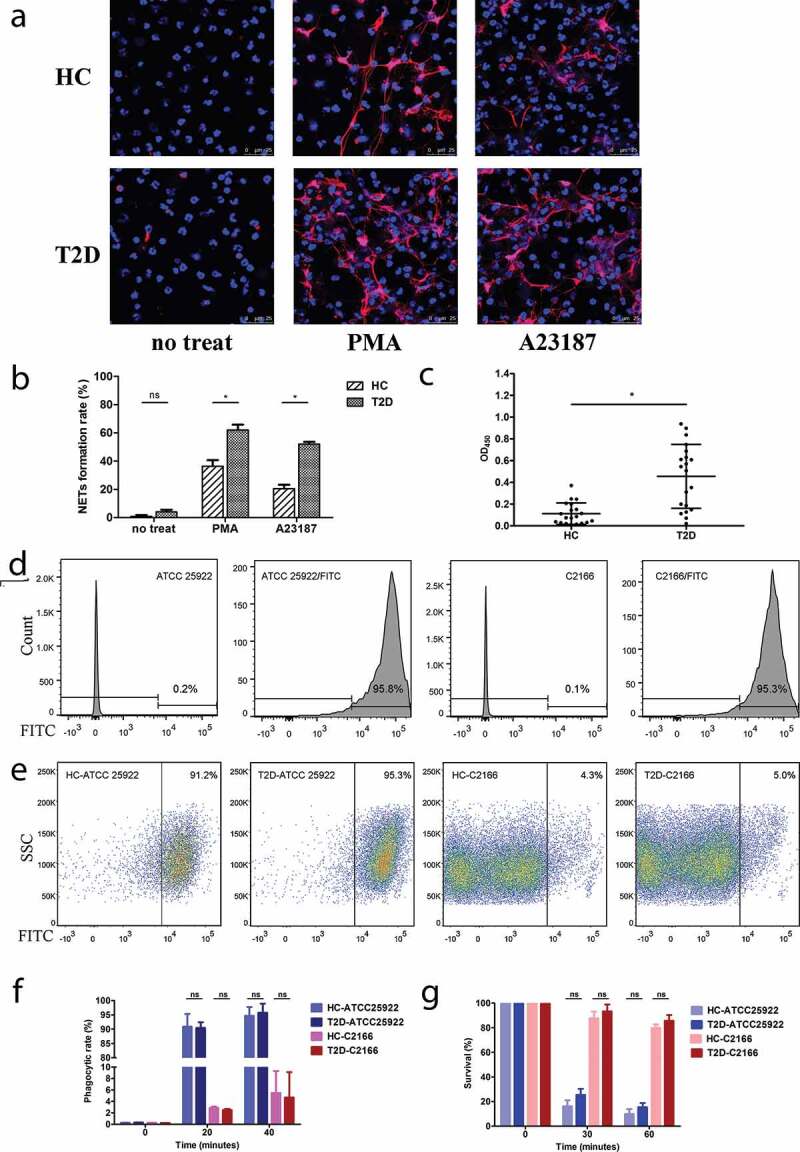
Representative immunofluorescence images (a) and quantification of the percentages of neutrophils undergoing NETosis (b) in the presence of PMA (20 nM) and A23187 (1 μM). Type 2 diabetes mellitus patients (T2D) neutrophil form more extracellular traps than healthy controls (HC) with the same stimulation. For the NETs induction, samples from five diabetics and five healthy volunteers were included. Neutrophils from each sample were induced by PMA or A23187 on three separate coverslips and six random fields were acquired per coverslip. On average, each field approximately contains 81 cells and each sample can count about 1400 cells. (c) Quantification of MPO-DNA complexes in serum samples of HC and T2D patients. (d-g) CR-hvKP C2166 showed highly resistant to neutrophilic phagocytosis using different assay methods. (d) *E. coli* ATCC 25922 and *K. pneumoniae* C2166 have a comparable efficiency of FITC labeling and more than 95% of the population can be stained effectively. (e) CR-hvKp C2166 displayed similar levels of resistance to phagocytosis by healthy and diabetic neutrophils after 40-min incubation. *E. coli* strain ATCC 25922 is susceptible to neutrophil phagocytosis and used as neutrophil phagocytosis function control in this study. (f) FITC-labeled bacteria detection based on flow cytometry; (g) CFUs recovery percentage based on DNase I treated neutrophil killing assays. ns, no significance; *p < 0.05: SSC, side scatter.

To clarify the discrepancy of the previous findings on the status of NETs formation in T2D [14,15], freshly isolated neutrophils from patients with T2D and HCs were stimulated with PMA or A23187 for 3 h at 37°C to induce NETosis. Compared to untreated neutrophils, treatment of PMA or A23187-induced excessive NETs formation in neutrophils from HCs as determined by the typical DNA filaments morphology with MPO (Figure 1(a)). Of interest, the PMA- or A23187-induced NETs formation was significantly increased in neutrophils from patients with T2D, compared to those from HCs (Figure 1(b)). The enhanced NETs formation in T2D was also confirmed by the significantly elevated levels of circulating MPO-DNA complexes (Figure 1(c)). Taken together, our findings confirmed previous observation that NETosis is enhanced in T2D and suggested that the susceptibility to CR-hvKP infection in T2D is not due to the deficient NETs formation.

### C2166 belongs to carbapenem-resistant hypervirulent K. pneuomiae dominant clone

To gain insight into the role of NETs in the host defense against CR-hvKP infection in T2D, we utilized a previously described clinical-derived CR-hvKP strain C2166 [4]. The strain was subjected to whole genome sequencing. Genomic characteristics on antimicrobial resistance and virulence were annotated and aligned with the genome of reference strain from the public database. C2166 belongs to ST11 (Clonal Group 258) and capsular type K64 *K. pneuomiae*, which is the most predominant CRKP clone in China and other Asian countries [2]. This strain carried multiple virulence genes encoding type 1 fimbriae, type 3 fimbriae, type 2 secretion system (T2SS), type 6 secretion system (T6SS), yersiniabactin, regulators of the mucoid phenotype (RmpA2) and aerobactin, which are known to be associated with hypervirulence in *K. pneumoniae* and also present in hvKP strain NTUH-K2044 and strain CR-hvKP4 [3]. Results of major virulence and antimicrobial resistance genes alignment are summarized in Table 1.

**Table 1.** Major virulence and antimicrobial resistance mechanisms were detected in carbapenem-resistant hypervirulent *K. pneumoniae* C2166 and counterpart reference strains using whole genome sequencing data.

Strains	MLST/K-type	Fimbriae	Secretion System (SS)	Siderophore	Regulators of the Mucoid Phenotype	β-lactamase
NTUH-K2044 (hvKP)	ST23/KL1	-	T2SS; T6SS;	Enterobactin; Yersiniabactin; Aerobactin; Salmochelin;	RmpA RmpA2	SHV-11;
CR-hvKP4 (CR-hvKP)	ST11/KL47	Type 1 Fimbriae; Type 3 Fimbriae;	T2SS; T6SS;	Enterobactin; Yersiniabactin; Aerobactin;	RmpA2	SHV-11; KPC-2 CTX-M-65;
C2166 (CR-hvKP)	ST11/KL64	Type 1 Fimbriae; Type 3 Fimbriae;	T2SS; T6SS;	Enterobactin; Yersiniabactin; Salmochelin; Aerobactin;	RmpA2 RmpA2	SHV-11; KPC-2 CTX-M-65;
HS11286 (CRKP)	ST11/KL103	-	T2SS; T6SS;	Enterobactin; Yersiniabactin;	-	SHV-11; KPC-2;

NTUH-K2044, ST23 hvKP reference strain; CR-hvKP4, ST11 CR-hvKP reference strain; C2166, ST11 CR-hvKP used in this study; HS11268, ST11 classical CRKP.

### CR-hvKp C2166 displayed similar levels of resistance to phagocytosis by healthy and diabetic neutrophils

Phagocytosis is one of the most efficient bactericidal mechanisms utilized by neutrophils [3]. To determine whether the increased susceptibility of CR-hvKp infection in T2D patients was due to defective phagocytosis in neutrophils, phagocytosis assay was performed on neutrophils from T2D patients and HCs using FITC-labeled C2166. Firstly, we determined that C2166 has a comparable FITC-labeling efficiency to the *E. coli* ATCC 25922 strain and more than 95% of the population can be stained effectively (Figure 1(d)). Consistent with previous observations that hvKP are resistant to neutrophilic phagocytosis, C2166 displayed significantly lower levels of phagocytic rate compared to the control strain ATCC 25922 (5.47% vs. 94.65%). Of interest, no significant differences in the levels of phagocytic rate were observed between neutrophils from T2D and neutrophils from HCs (Figure 1(e-f)). These observations were also corroborated by the DNase I treated neutrophil killing assays (Figure 1(g)) because DNase I could inhibit NET formation and disrupt NETs structure but no effect on phagocytosis function. These results suggest that the differences in susceptibility to CR-hvKP infections between patients with T2D and HCs are not ascribed to the phagocytosis defect in diabetes neutrophils.

### Diabetic NETs killing ability decreased markedly

**Figure 2.**
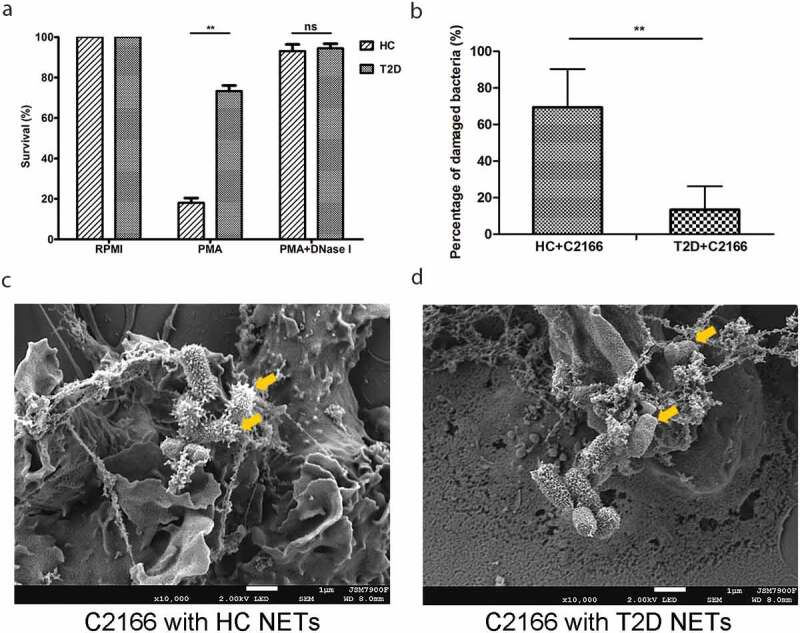
(a) Healthy controls (HC) and type 2 diabetes mellitus (T2D) patients NETs *in vitro* killing of CR-hvKP C2166. NETs were induced by 100 nM PMA for 3 h, then C2166 (1 × 10^6^ CFUs) were added and co-incubated for another hour. After cells lysing, C2166 were plated on LB agar and enumerated the next day. HC and T2D patients NETs were combined with C2166 and bacterial surface were analyzed under scanning electron microscope. The mean percentage of surface-damaged bacteria by HC and T2D NETs was compared and shown in (b). Representative images were presented in (c) and (d). **p < 0.01, ns: no significance.

NETs have been considered as a robust antimicrobial machinery by neutrophils for killing extracellular pathogens. The discrepancy between enhanced NETosis and increased susceptibility to CR-hvKP infections in T2D propelled us to investigate whether NETs in T2D were deficient in killing CR-hvKP. Neutrophils from T2D patients and HCs were isolated and *in vitro* induced to form NETs. Expectedly, incubation C2166 with NETs led to significantly decreased bacteria survival rates. Importantly, C2166 co-cultured with NETs from HCs displayed significantly lower survival rate compared to those cu-cultured with NETs from patients with T2D (Figure 2(a)). Specifically, after 1-h incubation with NETs, over 70% bacteria survived in NETs from T2D, while less than 20% bacteria survived in NETs from HCs. Further, treatment with DNase I abrogated NETs bactericidal capacity in killing CR-hvKP, suggesting that intact NETs structures are required to limit pathogen viability (Figure 2(a)). To better illustrate the difference of NETs killing ability between T2D patients and healthy control, we also measured the bacteria density after different co-incubation time (0, 0.5, 1.0, and 2.0 h). As shown in Supplementary Figure 1a, after 2-h NETs complex exposure, a higher bacterial survival was seen in T2D patients group than that of healthy control. Taken together, these data suggest that NETs play an important role in killing CR-hvKP and their bactericidal capacity to CR-hvKP are significantly impaired in T2D.

### NETs from patients with T2D displayed reduced damage to CR-hvKP surface

**Figure 3.**
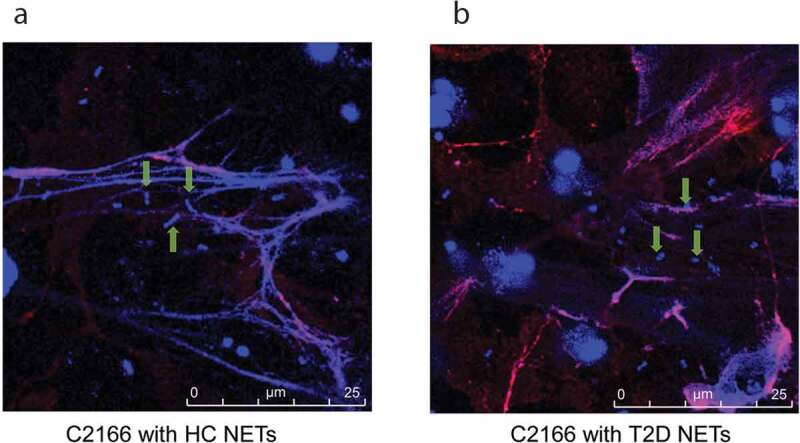
Healthy controls (HC) and type 2 diabetes mellitus patients (T2D) NETs can entrap a similar amount of C2166 strain. Green arrows indicate the ensnared bacteria within the NETs structure.

NETs bactericidal process initiates with trapping the pathogen into the web-like structure, followed by disrupting them with neutrophil-derived antimicrobial proteins. By utilizing immunofluorescence microscopy, we found that C2166 were trapped in NETs from patients with T2D to a similar extent with NETs from HCs (Figure 3). Generally, each NETs-like structure traped three to five bacteria in our experimental conditions, indicating NETs-mediated bacteria trapping is not impaired in T2D. We then employed the scanning electron microscope (SEM) to examine surface damage of C2166 co-cultured with either NETs from HCs or NETs from T2D. Consistent with previous observations that C2166 displayed lower survival rates in HCs-derived NETs conditions, SEM illustrated evident cell surface destruction of C2166 during their interaction with NETs from HCs, whereas C2166 remained relatively smooth and normal shape during their interaction with NETs from T2D (Figure 2(b-d)). As shown in Supplementary Figure 1b, sub-MICs of PGLa and polymyxin E have no impact on the growth of *K. pneumoniae*, while it markedly improved the killing activity of T2D NETs to a similar level of normal NETs. PGLa and polymyxin E have been documented to bind bacterial outer membrane and disturb the cell surface integrity. It indicated the deficient damage on cell surface is a possible reason for the impaired NETs killing by T2D patients. These results suggested that the impaired bactericidal activity of diabetic NETs to CR-hvKP is likely associated with the deficient surface damage caused by T2D patients NETs, which would contribute to the increased susceptibility to CR-hvKP infections in patients with T2D.

## Discussion

Multiple studies have suggested that patients with T2D display significantly increased risk of CR-hvKP infections [6,23]. In this study, we show that this elevated risk does not come from the differences in neutrophilic phagocytosis, as neutrophils from both HCs and patients with T2D failed to effectively phagocytose CR-hvKP. Instead, we demonstrate that the defective NETs-mediated killing of CR-hvKP strain contributes to the increased risk of CR-hvKP infections in patients with T2D. Specifically, we show that the impaired NETs-mediated killing in T2D is not due to the decreased NETs formation, as the neutrophils isolated from T2D patients exhibited enhanced NETs formation compared to healthy controls. Further, we demonstrate that the reduced NETs activity does not result from the trapping failure of CR-hvKP, but is due to the defect in bacteria surface destruction. Our data provide novel insight into the defective innate immune response against CR-hvKP in T2D, which sheds light on the design of novel NETs-based therapies and ultimately improve the therapeutic strategy against CR-hvKP infections in patients with T2D.

The pyogenic liver abscess is the most frequent invasive infection especially in diabetes mellitus patients. Hv-KP infections, which mainly cause pyogenic liver abscess, represent a considerable healthcare burden across Asian countries [6]. Even worse, hv-KP equipped with extensive antibiotic resistance, such as the CR-hvKP isolates, is increasingly being identified. Actually, clinicians have limited therapeutic approaches to treat the infections caused by this “superbug”. This warrants an improved understanding of innate immune components in this disease. Neutrophils play an essential role in the control of CR-hvKP infection, since these cells are attracted to the site of infection rapidly and eliminate invading pathogens by intracellular killing of phagocytosis and extracellular killing of NETs. HvKP strains have been shown to be highly resistant to phagocytosis in many previous studies [23–25]. Our studies also found that CR-hvKP are rarely engulfed (<5.5%) by neutrophils and phagocytosis killing significantly abrogated both in normal individuals and diabetes patients neutrophils.

To explain the impaired bacterial killing of diabetic neutrophil, we sought to determine the role of NETs exposed to extracellular CR-hvKP. Previous studies demonstrated that diabetes patients activate neutrophils to overproduce NETs which delayed the wound healing due to the potent pro-inflammatory activity [15,16]. We also confirmed that diabetes patients predispose peripheral neutrophil to release extracellular DNA traps *in vivo* and *ex vivo*. Because NETs were originally recognized as a host defense mechanism against a variety of microbes, then we test the diabetes NETs property of containing and killing pathogens *ex vivo*. Our results indicate that disabled NETs killing is not likely due to the absence of CR-hvKP within the NETs cage, the major reason is the direct bactericidal function delivered by diabetes patient neutrophil antimicrobial components in NETs is restrained significantly compared to healthy controls.

A previous study reported that the NETs killing to *K. pneumoniae* was not significantly impaired in T2D patients compared with healthy volunteers [19]. Considering the different *K. pneumoniae* strains and experimental conditions, the different phenotype we observed could be partially explained. Specifically, the study by Lee et al. used different hvKP strains (serotype K1/K2/K28) to induce neutrophils to spontaneously form NETs followed by a NETs killing test [19]. As described previously, PMA induced NETs were co-incubated with ST11 CR-hvKP strain in the present study, which guaranteed a similar level of NETs formation between T2D patients and HCs group.

Recently, it has been suggested that the role of NETs in different diseases is determined mainly by their disease-related protein load, which may be due to the alterations in the expression levels of neutrophil-associated proteins in distinct disease-specific microenvironments [26,27]. A recent proteomic study showed that 35 proteins were differentially expressed between diabetic patients with good glycemic control and those with poor glycemic control [28]. Of interest, some key antimicrobial components of NETs, including MPO, azurocidin (CAP37), and SA100A9, were downregulated in diabetic patients with poor glycemic control. These findings suggest association between impaired NETs killing and dysregulation of protein components in NETs among patients with poorly controlled diabetes. In future, we will investigate the molecular basis and downstream impacts of the difference in diabetes and normal NETs biological behaviors against CR-hvKP strain. It will be helpful to restore NETs killing activity in diabetes to combat against CR-hvKP and other pathogens infection.

In brief, our study revealed that the direct killing CR-hvKP strain by diabetes patient NETs was significantly abrogated compared to normal human NETs even invading pathogens have been restricted within the NETs structure. This result suggests that impaired NETs antimicrobial capacity render the diabetic host vulnerable to the development of CR-hvKP invasive infections.

## Supplementary Material

Supplemental Material
