# Supplementary figures and images for: Neutrophil extracellular traps (NETs)-mediated killing of carbapenem-resistant hypervirulent Klebsiella pneumoniae (CR-hvKP) are impaired in patients with diabetes mellitus
Source: Virulence. 2020 Aug 29;11(1):1122–30. doi: 10.1080/21505594.2020.1809325 (PMC7549946; doi:10.1080/21505594.2020.1809325)

Figure S1

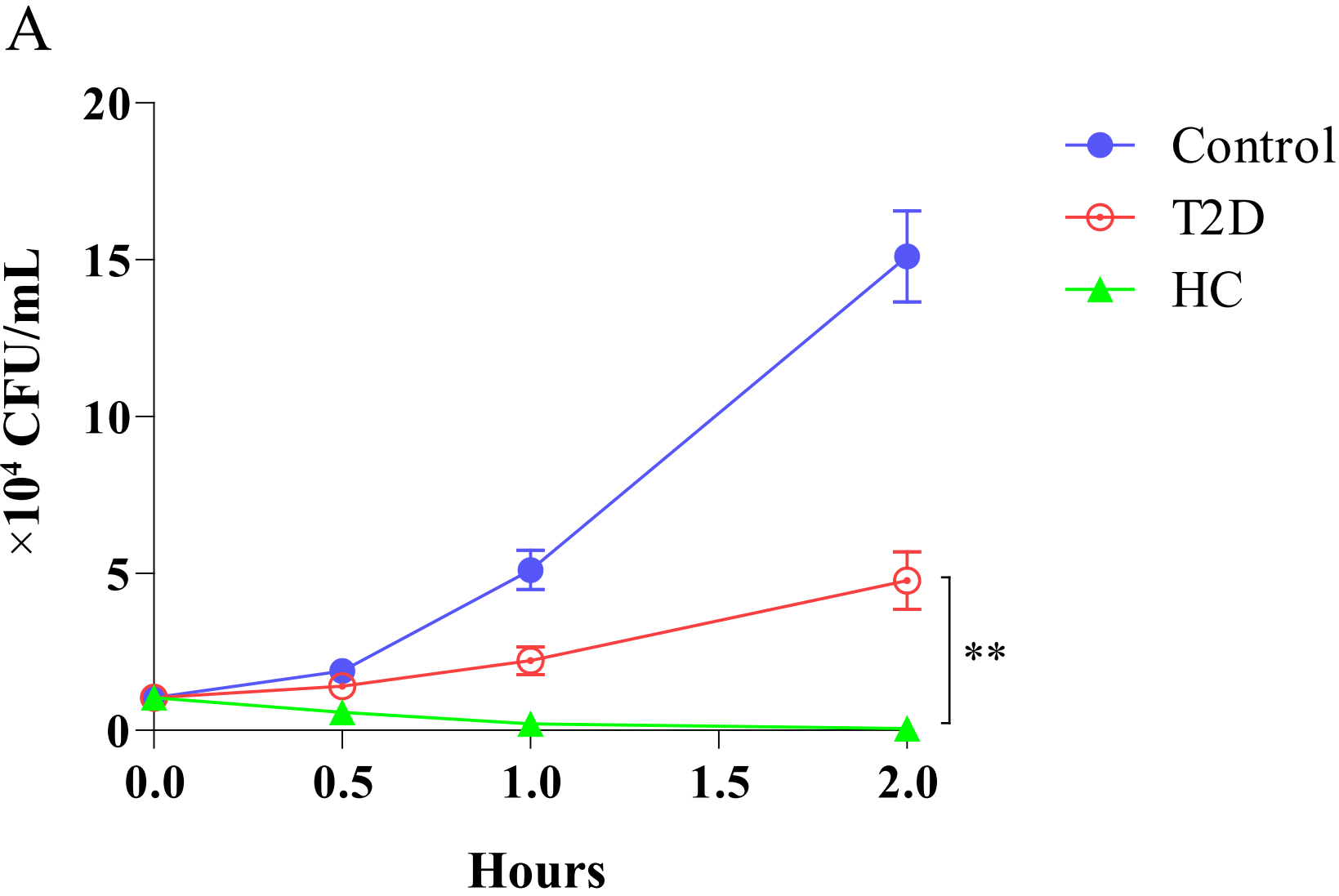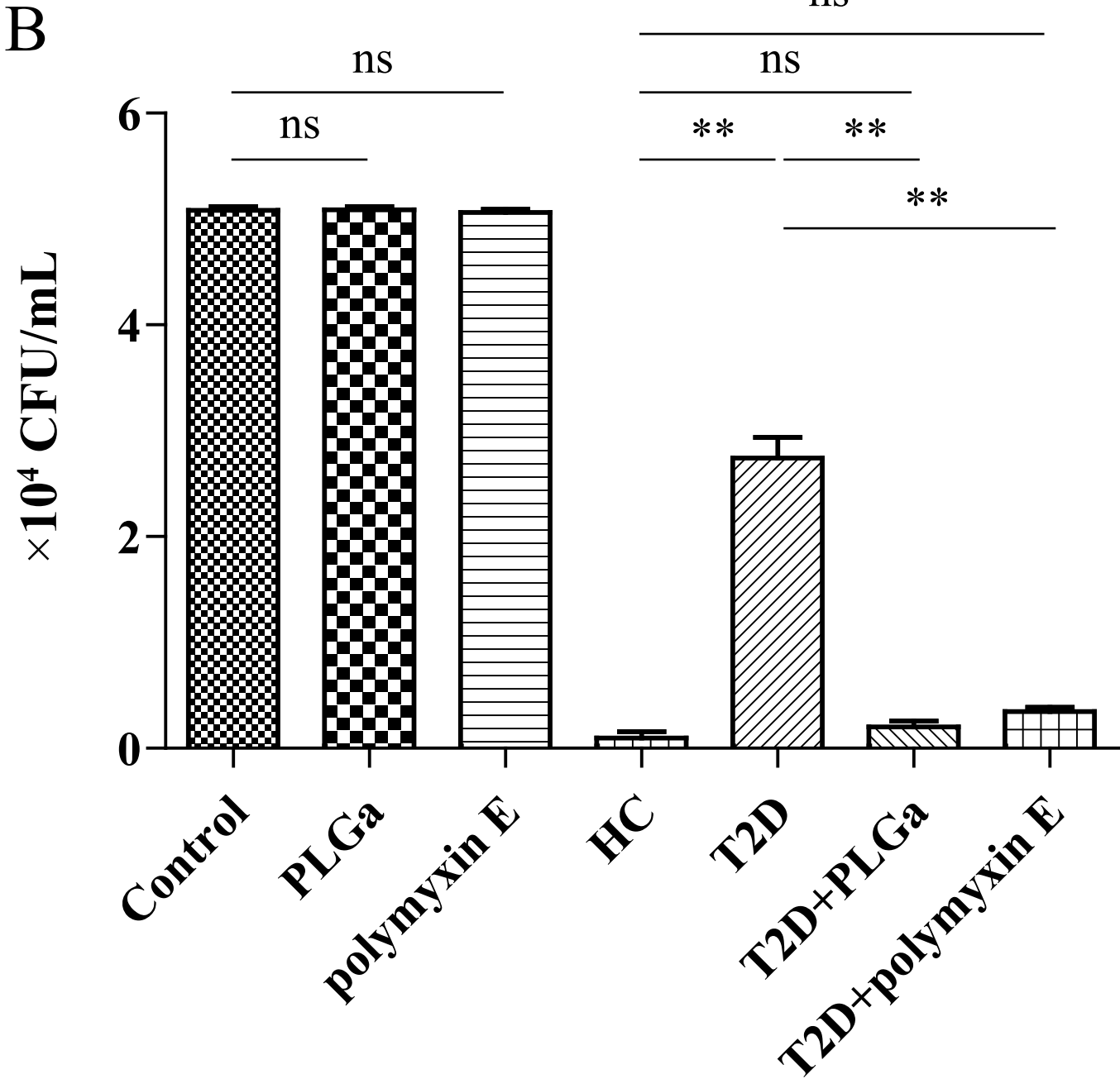

Supplement: Supplemental Material [file KVIR_A_1809325_SM9711.pdf]
